# Early Family Intervention for Youth at Risk for Bipolar Disorder: Psychosocial and Neural Mediators of Outcome

**DOI:** 10.2174/1570159X21666230111120817

**Published:** 2023-05-12

**Authors:** David J. Miklowitz, Marc J. Weintraub, Patricia D. Walshaw, Christopher D. Schneck, Kiki D. Chang, John Merranko, Amy S. Garrett, Manpreet K. Singh

**Affiliations:** 1 Semel Institute for Neuroscience and Human Behavior, University of California, Los Angeles, Los Angeles, CA, USA;; 2 School of Medicine, University of Colorado Anschutz Medical Campus, Aurora, CO, USA;; 3 Private Practice, 2460 Park Blvd, Suite 6 Palo Alto, CA 94306 USA;; 4 Department of Psychiatry, University of Pittsburgh Medical Center, Pittsburgh, PA, USA;; 5 Department of Psychiatry, University of Texas, Health Science Center at San Antonio, San Antonio, TX, USA;; 6 Department of Psychiatry, Stanford University, Stanford, CA, USA

**Keywords:** Neuroimaging, family therapy, psychosocial, mediators, emotion regulation

## Abstract

**Background:**

The impairing neurodevelopmental course of bipolar disorder (BD) suggests the importance of early intervention for youth in the beginning phases of the illness.

**Objective:**

We report the results of a 3-site randomized trial of family-focused therapy for youth at high-risk (FFT-HR) for BD, and explore psychosocial and neuroimaging variables as mediators of treatment effects.

**Methods:**

High-risk youth (<18 years) with major depressive disorder or other specified BD, active mood symptoms, and a family history of BD were randomly assigned to 4 months of FFT-HR (psychoeducation, communication and problem-solving skills training) or 4 months of enhanced care psychoeducation. Adjunctive pharmacotherapy was provided by study psychiatrists. Neuroimaging scans were conducted before and after psychosocial treatments in eligible participants. Independent evaluators interviewed participants every 4-6 months over 1-4 years regarding symptomatic outcomes.

**Results:**

Among 127 youth (mean 13.2 ± 2.6 years) over a median of 98 weeks, FFT-HR was associated with longer intervals prior to new mood episodes and lower levels of suicidal ideation than enhanced care. Reductions in perceived family conflict mediated the effects of psychosocial interventions on the course of mood symptoms. Among 34 participants with pre-/post-treatment fMRI scans, youth in FFT-HR had (a) stronger resting state connectivity between ventrolateral PFC and anterior default mode network, and (b) increased activity of dorsolateral and medial PFC in emotion processing and problem-solving tasks, compared to youth in enhanced care.

**Conclusion:**

FFT-HR may delay new mood episodes in symptomatic youth with familial liability to BD. Putative treatment mechanisms include neural adaptations suggestive of improved emotion regulation.

**Clinical Trial Registration Information:**

Early Intervention for Youth at Risk for Bipolar Disorder; https://clinicaltrials.gov/; NCT01483391.

## 1 INTRODUCTION

Bipolar disorder (BD) can be recognized in childhood and adolescence, typically in the form of depressive symptoms or episodes, subsyndromal mania or hypomania, anxiety, and mood instability [1-3]. High-risk symptom presentations in combination with a family history of BD significantly increase the risks for children to convert to syndromal BD I or II over 5-8 years [3, 4]. High-risk youth have levels of depressive symptom severity and functional impairment that are comparable to levels observed in youth with full-threshold BD [5, 6].

The developmental pathways to the onset of BD in vulnerable youth are being clarified in genetic, neuroimaging, and psychosocial studies. Certain genes appear to mediate the relation between having a parent with BD and an offspring’s likelihood of developing BD [7]. Healthy offspring of parents with BD have distinct prefrontal, striatal, and limbic function and connectivity at rest [8] and while processing face emotions [9], compared to healthy offspring of depressed and non-depressed parents. Furthermore, youth at high risk for BD are often exposed to high levels of family conflict, low levels of family cohesion, and stressful life events [10-13]. These lines of evidence suggest that a neurogenetic predisposition for developing BD may precede symptom onset and interact with environmental stressors, leading to symptom emergence and progression in vulnerable offspring.

Despite these advances, it is unclear what parents or treating clinicians should do when behavioral or familial risk factors suggest a trajectory toward illness onset in a child or teen with early symptoms. Intervening with pharmacotherapy may lead to reductions in subthreshold symptoms in high-risk youth [14]. It is also associated with adverse events, such as excessive agitation, weight gain, or metabolic disorders [15-17]. High-risk youth appear to require more than pharmacotherapy to manage mood symptoms that, for some, represent the early phases of the disorder.

These challenges have inspired the nascent work on preventative psychosocial interventions for youth at risk for BD. Preventative psychosocial interventions must have efficacy in minimizing early-onset symptoms as well as preventing future episodes, either alone or in combination with pharmacotherapy. Ideally, they should have an impact on the child’s reaction to stressors that may aggravate symptoms. In prior randomized trials, we have shown that a psychoeducational intervention called family-focused therapy (FFT), when combined with adequate pharmacotherapy, is effective in delaying recurrences and reducing symptom severity in adults with BD I and II [18]. In adolescents with BD I and II, FFT and pharmacotherapy were observed to mitigate disease progression by reducing symptom severity and enhancing psychosocial functioning over 2 years when compared to brief psychoeducation and pharmacotherapy [19, 20].

We reasoned that FFT could be adapted as an early intervention for youth at high risk for conversion to BD. FFT for high-risk youth (FFT-HR) begins when a youth with a family history of BD shows subthreshold manic or sub- or full threshold depressive symptoms. In 12 sessions over 4 months, clinicians provide the child and family members with psychoeducation about the nature, causes and prevention of mood disorders, followed by communication and problem-solving skills training to decrease family conflict and increase cohesion. In a pilot randomized trial of FFT-HR in 40 high-risk children, family treatment plus pharmacotherapy was more effective than brief education plus pharmacotherapy in decreasing time to recovery from mood episodes, increasing time in symptom remission, and reducing hypo/mania symptoms over one year [21]. A subgroup of 12 trial participants who were scanned using fMRI at pre- and post-treatment demonstrated symptom improvement over 4 months that was correlated with increased dorsolateral prefrontal cortex (DLPFC) activation during emotional face processing [22].

This article describes clinical outcomes from a larger-scale randomized trial of high-risk youth recruited from three study sites. We compared FFT-HR with a duration-matched, standardized psychoeducational therapy (enhanced usual care, or EC) on symptomatic outcomes (*i.e*., time to new mood episodes, symptom severity over time) in high-risk youth. In addition to psychotherapy, participants were offered best-practice pharmacotherapy using a treatment algorithm [23]. The primary results of this trial have been reported elsewhere [24-26]. Here, we explore the psychosocial and neural factors that have emerged as mediators of the relation between the family intervention and symptom change over a median of 2 years of follow-up. We explore two key questions: (1) what are the effects of FFT-HR compared to EC on family relationship variables, and do changes in these variables explain the association between treatment condition and symptomatic outcomes? (2) Are these treatments associated with different patterns of change in neural circuits relevant to processing emotions or solving problems, processes that are believed to be altered in the pre-symptomatic and early phases of BD [27, 28]?

To comprehensively investigate the engagement of progressively higher-order neural systems targeted by early intervention, we used three methods of assaying changes in corticolimbic circuits from before to after FFT-HR or EC: in the resting state, during a facial emotion processing task, and during a problem-solving task. We hypothesized that key intrinsic connectivity patterns associated with known bipolar networks, as shown in the resting state, would change with treatment, as would amygdala activation during face emotion processing. Further, because amygdala hyperactivity and diminished prefrontal inhibitory control have been associated with mood instability in BD [29], we challenged high-risk youth with a novel in-scanner family problem solving task designed to evaluate the effects of psychosocial intervention on mood instability. Clarifying the relations between changes in corticolimbic targets and the trajectories of mood symptoms that follow may clarify the mechanisms by which psychosocial interventions alter 2-year outcomes in high-risk youth.

## 2 METHODOLOGICAL APPROACH: DESIGN OF THE TRIAL

### 2.1 Participants

The trial was advertised through pediatricians, social media platforms, and study flyers posted at specialty clinics at the University of California Los Angeles (UCLA) Semel Institute, Los Angeles, CA; the University of Colorado Anschutz Medical Campus in Aurora, CO; and the Stanford University Department of Psychiatry, Stanford, CA, USA. We enrolled youth who met the following criteria: (1) age between 9 and 17 years, 11 mos.; (2) met DSM-5 criteria for otherwise specified BD (OSBD) or major depressive disorder, based on the Kiddie Schedule for Affective Disorders and Schizophrenia, Present and Lifetime interview for DSM-IV [30] and later, DSM-5 [31]; (3) had at least one first- or second-degree relative with a lifetime DSM-5 diagnosis of BD I or II, based on direct interviews of relatives using the MINI-International Neuropsychiatric Interview [32]. When relatives were unavailable, we used reports from available relatives on the Family History Screen [33]; and (4) had active mood symptoms at the time of assessment, as indicated by a Young Mania Rating Scale [34] (YMRS) score > 11 in the prior week or a Children’s Depression Rating Scale, Revised [35] (CDRS-R) score > 29 over the prior 2 weeks. We adopted the Course and Outcome of Bipolar Youth criteria [36] for defining OSBD: distinct periods of abnormally elevated, expansive or irritable mood plus two (three, if irritable mood only) DSM symptoms of mania that caused a change in functioning, lasted ≥ 4 hours in a day, and occurred for a total of 10 or more days in the child’s lifetime.

A child psychiatrist conducted a separate evaluation to provide a second opinion on whether the proband met the study’s eligibility criteria. Probands who consented and were deemed study-eligible were offered medication management with this or another study-affiliated psychiatrist, who based treatment recommendations on medication guidelines developed for this population [23]. The majority of youth (60%) received mood stabilizers or second-generation antipsychotic agents while enrolled in the study.

### 2.2 Random Assignment to Psychosocial Interventions

Once eligible, children and families were randomly assigned to FFT-HR or enhanced usual care (EC) using a dynamic allocation procedure [37] that balanced treatment groups within sites on diagnosis, age, and baseline pharmacotherapy (mood stabilizers or antipsychotics *vs*. neither). FFT-HR consisted of 12 sixty-minute sessions in four months (8 weekly, 4 biweekly). Family sessions, which included the child, the parent(s), and whenever possible, siblings, focused on (1) psychoeducation about managing mood disorders, including assisting the family in recognizing the early warning signs of hypomanic, manic or depressive episodes; the role of stress in eliciting symptoms; and development of a mood management plan (a written list of early warning signs, antecedent stressors, and preventative strategies); (2) communication enhancement training, consisting of in- and between-session rehearsal of relational skills including active listening, offering positive or negative feedback, making requests for change in another’s behavior, and communication clarity; and (3) problem-solving skills training, in which families learned to define areas of conflict, generate possible solutions, evaluate their advantages and disadvantages, and choose one or a set of solutions to implement in the forthcoming week. The EC condition, while of the same duration as FFT-HR (4 months), consisted of 3 weekly 60-minute family psychoeducation sessions followed by 3 monthly individual psychoeducation sessions focused on implementing a mood management plan. Clinicians were trained to administer FFT-HR and EC in a study launch meeting and supervised monthly throughout the study. Mean Therapist Competence and Adherence Scale ratings [38] indicated consistently high levels of fidelity to both protocols.

### 2.3 Interview-based Clinical Outcome Assessments

Independent evaluators who were unaware of treatment assignments conducted interviews with the youth and at least one parent at the time of randomization (covering the 4 prior months), every 4 months in year 1, and then every 6 months for up to 4 years (median follow-up of 98 weeks, range 0-255 weeks). To quantify the severity and polarity of symptoms at each follow-up interview, evaluators administered the YMRS (hypo/mania) and CDRS-R (depression) interviews covering the prior 1- and 2-week intervals, respectively. Next, evaluators rated each week of the preceding 4- or 6-month interval using the Adolescent Longitudinal Interval Follow-up Evaluation (A-LIFE) Psychiatric Status Ratings (PSRs) [39]. The weekly PSRs are 1 (asymptomatic) to 6 (fully syndromal, severe) point scales of severity and impairment, with separate scales covering depression, mania, and hypomania. The weekly PSR for depression ranged from 1 (symptoms absent) to 6 (severe symptoms), whereas the weekly PSRs for mania and hypomania were combined into a single 8-point hypo/mania scale ranging from 1 (no symptoms) to 6 (syndromal hypomania), with scores of 7 and 8 reserved for severe or extremely severe mania.

PSR data were used to define the time to recovery from pretreatment symptoms (all PSR scales ≤ 2 (mildly symptomatic) for ≥ 8 consecutive weeks) and among those who recovered during follow-up, time to new mood episodes or recurrences (≥ 2 weeks with PSR depression ratings of 4 (syndromal, moderate severity) or higher, or ≥ 1 week PSR hypo/mania ratings of 5 (syndromal) or higher). Interrater reliabilities for weekly depression and hypo/mania PSRs were 0.88-0.99 (intraclass rs) across raters at three sites.

### 2.4 Self-report Assessments

To expand our understanding of the symptom and functional impact of psychosocial interventions, high-risk probands completed a battery of questionnaires at intake and at each 4-6 months follow-up: the 15-item Suicidal Ideation Questionnaire, Junior Version [40]; the Social Adjustment Scale-Self Report [41] assessing functioning in family relationships, peer relationships, and school; and the Conflict Behavior Questionnaire [42] (CBQ), a 20-item true/false scale that assesses the degree of aversive communication, frustration, empathy, and relationship quality in child/parent dyads over the prior 3 months. The 20-item parent-rated Children’s Affective Lability Scale [43] (CALS) was used to measure the child’s mood instability (ongoing, frequent, and unpredictable shifts in emotional states) over the prior 3 months, using 1 (behavior never or rarely occurs) to 5 (≥ 1 times a day) scales of frequency. Example items include: “Has bursts of silliness for little or no apparent reason”, or “Suddenly loses his/her temper when you would not expect it”.

### 2.5 Statistical Analyses: Comparisons of Treatment Groups for Symptomatic Outcomes

Details of the statistical modeling are described elsewhere [24, 26]. Briefly, we compared youth in the FFT-HR and EC groups in terms of the number of weeks from random allocation to the beginning of a period of recovery from pre-randomization symptoms. Then, among those who recovered, we compared treatment effects on time to newly emergent mood episodes. We obtained Kaplan-Meier estimates of the survival curves [44] for each treatment condition and used the log-rank procedure to test the overall effects of the treatment group.

Using repeated measures mixed-effect models, we examined the main and interactive effects of treatment (FFT-HR versus EC) and study assessment visit (every 4 months in year 1 and every 6 months thereafter) as predictors of depression or hypo/mania (mean weekly PSR) severity scores. In mediational analyses, we examined changes in family functioning as measured by the child’s reports on the Conflict Behavior Questionnaire (CBQ) and the Social Adjustment Scale-Self Report as mediators of the association between treatment condition and PSR scores from baseline to post-treatment and end of follow-up. To model mediation effects, PSR scores at follow-up were regressed on treatment group and youth’s CBQ or Social Adjustment Scale scores from the prior 4-6 month assessment interval, with random intercepts and slopes fitted within-subject. Indirect effects were estimated and bootstrapped with 1000 iterations to test the effects of the treatment group on PSR scores *via* the mediator, lagged CBQ or Social Adjustment scores. All submodels within the mediation model included study visit as a covariate. Mediational analyses were conducted using R 4.0.3 (https://www.R-project.org/).

### 2.6 Neuroimaging Hypotheses and Protocol

We examined changes in neural functioning from before to after 4 months of psychosocial treatment using functional magnetic resonance imaging (fMRI). Youth were scanned only if they did not have MRI contraindications (*e.g*., metal in the body) or orthodontic braces. Neuroimaging was only conducted at the UCLA and Stanford sites (n = 83) because of the lack of a comparable scanner environment at the Colorado site. At UCLA and Stanford, the same proportion of probands was scanned in each treatment group, with whole-brain analyses statistically adjusted for the effects of the site (Fig. **S1**).

Broadly, we hypothesized that intrinsic networks and neural network function subserving emotional control and problem-solving would be enhanced by FFT-HR compared to EC. Specifically, during rest, we hypothesized that FFT-HR would increase cortico-limbic connectivity to a greater extent than EC and that treatment-related changes in connectivity would correlate with pre- to post-treatment improvements in symptom severity. During emotion processing, we hypothesized that youth in FFT-HR would show greater reductions in amygdala activation and greater increases in DLPFC and ventrolateral prefrontal cortex (VLPFC) activation compared to those in EC. Further, we hypothesized that decreases in amygdala activation and increases in DLPFC activation would be associated with symptom improvement over the pre- to post-treatment interval. During problem solving, we hypothesized that changes in corticolimbic activation would correspond with changes in mood instability, which is frequently reported by parents and probands when the latter are challenged with daily complex executive functioning tasks, such as addressing conflicts within the family.

#### 2.6.1 Neuroimaging Analyses

Resting-state functional connectivity analyses aimed to examine changes in task-free correlational networks from pre- to post-treatment. We examined (a) default mode networks known for self-control and rumination during depression and (b) salience networks responsible for emotional expression. Group independent component analysis after automatic detection and removal of motion-related artifacts [45] (ICA-AROMA) was performed on resting state scans followed by dual regression [46]. The Multivariate and Repeated Measures toolbox [47] was used to test for changes in connectivity within 7 bipolar-relevant network components referenced from the literature [48, 49].

Second, emotion processing was evaluated using an implicit emotion perception task, in which subjects viewed photographs of young adult faces with happy, fearful, or calm expressions. Subjects were instructed to press a button to indicate the gender of each face, intending for the neural processing of emotion to be implicit. This task has been shown to reliably activate the amygdala in healthy youth and adults [50] and reveals amygdala hyperactivity in youth with BD [51-53] and at high risk for developing BD [54-56]. In our pilot FFT-HR trial, high-risk youth who completed this task before and after psychosocial treatments showed increases in DLPFC activity that correlated with symptom improvement [22]. A mixed-effects model was used to examine the main effects of and interactions between group (FFT-HR, EC) and time (pre- and post-treatment) on changes in neural activation during emotion processing, using a voxel-wise analysis of changes across the whole brain (a more conservative approach to hypothesis-testing than region-of-interest analyses). As the data showed similar activation across facial expressions, these analyses were run for all facial expressions combined (happy, fear, calm).

Finally, the youth engaged in a novel problem-solving task that involved viewing both family-oriented problems (*e.g*., “my mom and I argued about video games”) and non-family problems (*e.g*., “I have a paper due tomorrow”) which were personally relevant to each participant. Each trial of the task had two components: 1) briefly viewing a statement of the problem and then imagining it happening, and 2) mentally devising a way to solve the problem. Components of the task were analyzed separately for family and non-family problems. In statistical modeling, we examined how pre-post changes in neural activity correlated with pre-post changes in the child’s mood lability (using parent-rated CALS scores). Whole-brain mixed effects analyses were used to compare pre-to post-treatment changes in the FFT-HR *vs*. EC treatment groups, using change in CALS scores as a covariate of interest. Thresholding and correction for multiple comparisons for all analyses were achieved using nonparametric permutation testing with 5000 iterations, with a cluster-setting threshold of p=.001 and family-wise error (FWE) correction of p < .05 at the cluster level.

## 3 RESULTS

### 3.1 Clinical Outcomes

The sample characteristics are described in Table **1** (see [24] for Consort Diagram). Of 127 youth who were randomized to psychosocial treatments (mean 13.2 ± 2.6 years, 82 (64.6%) female), 90 (70.9%) symptomatically recovered from their pre-randomization mood symptoms, 23 did not recover, and 14 withdrew shortly after randomization. New mood episodes during the follow-up were observed in 71 (78.9%) of these 90 youth, the majority of which (77.8%) were episodes of major depression. Although there were no differences between treatment groups in time to recovery, youth in FFT-HR had longer intervals between symptom recovery and the next prospectively observed mood episode than youth in EC (log-rank χ2 = 5.44; *P* = .02; HR, 0.55; 95% CI, 0.48-0.92), and longer intervals from randomization to the next mood episode (χ2 = 4.44; *P* = .03; hazard ratio, 0.59; 95% CI, 0.35-0.97) [24].

The FFT-HR and EC groups were matched in treatment duration (4 months) but not in the number of sessions (12 *vs*. 6). In a Cox Proportional Hazards model, we examined the association between the treatment group and time to new mood episodes with the number of therapy sessions covaried. This model indicated that the rate of new episodes was lower in FFT-HR compared to EC (log-rank χ2(1) = 5.76; *P* = .016; HR, 0.33; 95% CI, 0.13-0.81), but there was no independent effect of therapy visits (log-rank χ2 = 1.90; *P* = .17; HR, 1.11; 95% CI, 0.96-1.28). This result suggests that observed differences between the two treatment groups were not solely due to group differences in the number of sessions.

There was no evidence that the treatment delayed or prevented the onset of bipolar I or II disorders, which was observed in 18 of the 113 cases (15.9%) with at least one follow-up assessment. Depressive symptoms were far more common during follow-up than symptoms of hypo/mania, with about half (50.8%) of the sample experiencing a course characterized by persistent depressive symptoms, 16.5% experiencing an ongoing, moderately symptomatic course, and one-third (32.5%) experiencing a significantly improving course over time [57]. These course patterns are similar to those observed among adults and adolescents with BD I or II disorders [58-61].

### 3.2 Psychosocial Variables in the Pathway from Treatment to Symptomatic Outcomes

What psychosocial factors might explain the effects of FFT-HR on the symptomatic course of youth with high-risk phenotypes? In a recent article [25], we reported that youth who had higher levels of suicidal ideation at baseline (based on the Suicidal Ideation Questionnaire) and who were randomly assigned to FFT-HR had lower levels of (and fewer weeks of) suicidal ideation at follow-up, and longer intervals without suicidal behavior compared to youth with high baseline levels of suicidal ideation who were treated with EC. Participants in FFT-HR reported less conflict behavior with mothers over an average of 2 years compared to participants in EC. Further, reductions in youth’s perceptions of conflict with mothers anticipated later reductions in suicidal ideation to a greater extent in FFT-HR compared to EC [25].

We observed a similar pattern in a separate analysis [26] using youth’s family functioning ratings on the Social Adjustment Scale-Self Report instrument as the putative mediator. First, family, social, and school functioning each improved over 24 months of follow-up, but there were greater improvements in youth’s ratings of family functioning (*i.e*., openness of communication, levels of conflict) in FFT-HR than in EC over time. Second, the relationship between treatment condition and changes in depressive symptoms from pre- to post-treatment and from pre-treatment to 24-month follow-up were mediated by changes in youth’s ratings of family functioning [26]. Thus, changes in youth’s perceptions of family functioning partially explained the pathways between treatment with FFT-HR and symptomatic improvements over 2 years.

### 3.3 Neuroimaging Results

What neural factors might explain the effects of FFT-HR (compared to EC) on the symptomatic course of youth with high-risk phenotypes? Out of 72 participants (out of a possible 83 at UCLA and Stanford) who received neuroimaging scans at baseline, 40 were scanned again after the 4-month interventions; usable resting state data were available for 34 (Fig. **S1**). All 40 participants produced usable repeat scan data for the emotion processing task, and 38 produced usable data for the problem-solving task.

Table **2** describes the clinical and demographic features of the 34 youth with usable pre-/post-treatment data across all three scanning tasks. This subsample did not differ from the participants who were not scanned (n = 93) on baseline levels of depression or mania/hypomania, diagnosis (other specified BD *vs*. major depressive disorder), gender, ethnicity, or race. The participants who were scanned were somewhat older (mean 13.98 ± 2.64 yrs.) than those who were not scanned (mean 12.93 ± 2.51 yrs.; f [1, 125] = 4.22, p = 0.04). In the scanned subsample, youth in FFT-HR (n=16) had longer intervals prior to new mood episodes (92.55 ± 13.70 weeks) compared to youth in EC (n=18; 65.50 ± 8.96 weeks), as observed in the full sample. Although this difference did not reach significance in the scanned sample (χ^2^(1) = 2.16, p=0.14), the hazard ratio was similar (0.55, 95% confidence interval, 0.21-1.45) to the hazard ratio in the full sample (HR, 0.59; 95% CI, 0.35-0.97).

#### 3.3.1 Neuroimaging, I: Changes in the Resting State

Examining interactions between the treatment group (FFT-HR, n=17 *vs*. EC, n=17) and time (pre *vs*. post, covarying scanner site, age, and sex), with family-wise error correction for multiple comparisons at the cluster level, did not yield any significant clusters in expected salience networks. There were, however, group differences in connectivity between the ventrolateral prefrontal cortex (VLPFC) and default mode network (DMN) components relevant to emotional expression and control at rest [46]. Results showed a significant group-by-study visit interaction on the connectivity between VLPFC and anterior default mode network (aDMN) (t=3.33, 95% CI (0.27, 1.14), p=.002). In this interaction, the FFT-HR group showed a significant increase in connectivity between right VLPFC and aDMN from pre- to post-treatment (t= 3.04, 95% CI (.16, .76), p=.003), whereas the EC group showed no significant change in connectivity over time (t= -1.63, 95% CI (-.50, .05), p=0.11). Neither primary diagnosis nor medication class exposures at baseline (*e.g*., presence/absence of antipsychotics, anticonvulsants, psychostimulants or antidepressants) predicted treatment-related changes in VLPFC-aDMN connectivity.

**Fig. (1):**
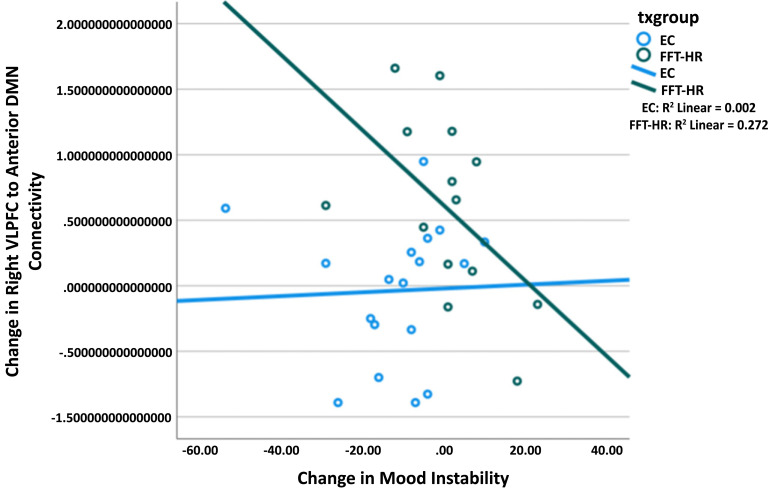
Neural changes during resting state. **Legend**: Right ventrolateral prefrontal cortex (VLPFC) connectivity with the anterior default mode network (DMN) is positively correlated with improvements in parent-rated Children’s Affective Lability Scale (mood instability) from pre to post-treatment in the FFT-HR group (p = .04) but not in the EC group (p = .97). The model adjusted for age, sex, site, and the number of days in treatment. FFT-HR = family focused therapy; EC = enhanced care.

Adjusting for age, sex, and scanner site, post-minus pre-treatment changes in anterior DMN connectivity were significantly correlated with post-minus pre-treatment changes in Children’s Depression Rating Scale scores in the FFT-HR group (r= -0.71 p=0.006) but not in the EC group (r= -0.07; p=0.82; Fisher’s *r*-to-*z*, z=-2.17, p=0.015). Further, after adjusting for age, sex, site, and the number of days in treatment, post-minus pre-treatment changes in right VLPFC and aDMN connectivity were significantly correlated with post-minus pre-treatment changes in overall CALS mood instability scores in the FFT-HR group (r=-0.64 p=0.04) but not in the EC group (r= 0.01; p=0.97; Fisher’s r-to-z, z=-2.03, p=0.021). (Fig. **1**). There was no association between connectivity changes and Young Mania Rating Scale scores within the FFT-HR or EC groups (all ps>0.05).

#### 3.3.2 Neuroimaging, II: Treatment-related Changes in Emotion Perception Using the Facial Expression Task

**Fig. (2):**
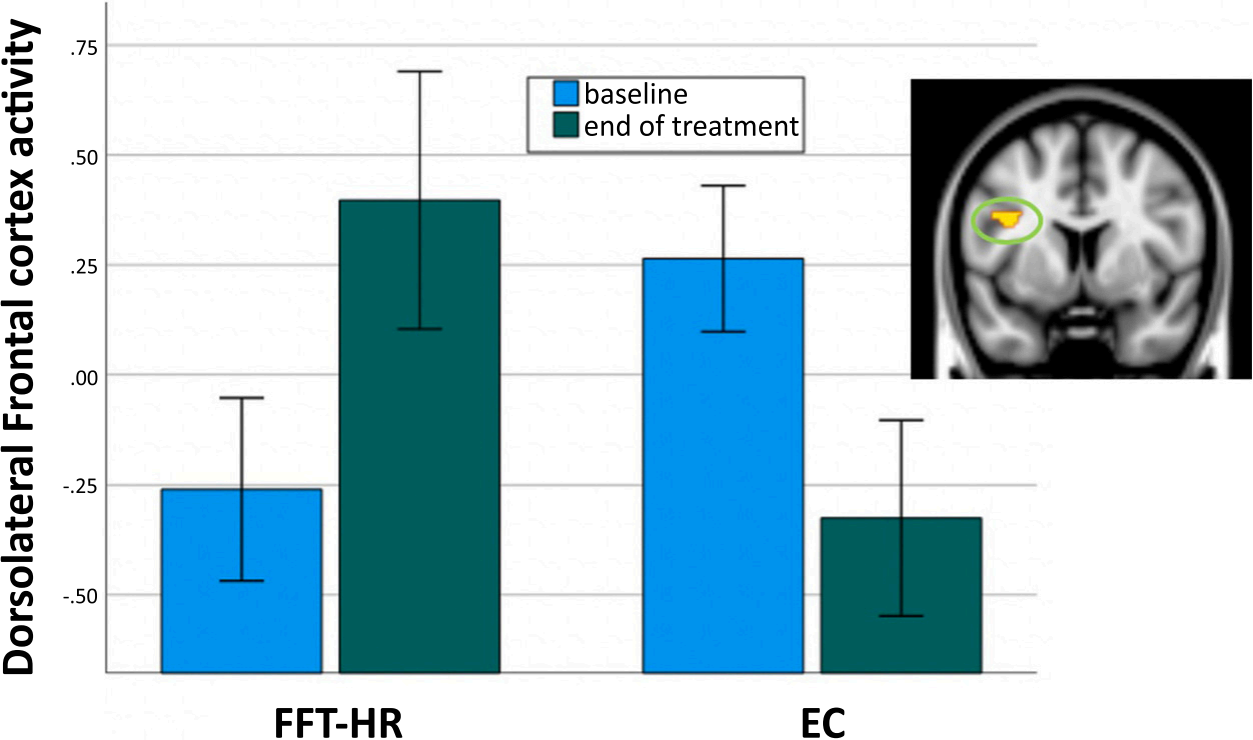
Neural changes during facial emotion processing. Legend: Significant group x time interaction in the left dorsolateral frontal cortex (DLPFC), indicating treatment group differences in longitudinal change from pre- to post-treatment, as measured by functional MRI during the emotional faces task. The bar graph depicts the mean contrast value in the cluster that is circled on the brain image. FFT-HR = family focused therapy; EC = enhanced care.

Twenty youth in FFT-HR and 20 in EC were included in the whole brain voxel-wise analysis of pre- to post-treatment neural changes during face emotion processing [62]. Results from the mixed effects analysis indicated a significant group x time interaction (p<.05, family-wise error correction for multiple comparisons at the cluster level). Follow-up within-group t-tests showed that DLPFC responses to faces increased from pre/post-treatment in the FFT-HR group (t = 3.8, *p* = .001) but decreased in the EC group (t = 3.2, *p* = .005) (Fig. **2**). For the main effect of time (summed across both treatments), decreasing levels of activation in the amygdala and hippocampus were correlated with pre- to post-treatment improvement in Young Mania Rating Scale scores (rho = −.41, *p* = .008), whereas increasing activation in the DLPFC was correlated with pre- to post-treatment improvements in Children’s Depression Rating Scale scores (rho = .49, *p* = .001) [62]. The presence or absence of antipsychotics, anticonvulsants, psychostimulants or antidepressants did not yield significant changes in treatment group effect sizes, nor did covarying the age of the child.

#### 3.3.3 Neuroimaging, III: Changes in Neural Activity During a Problem-solving Task

**Fig. (3):**
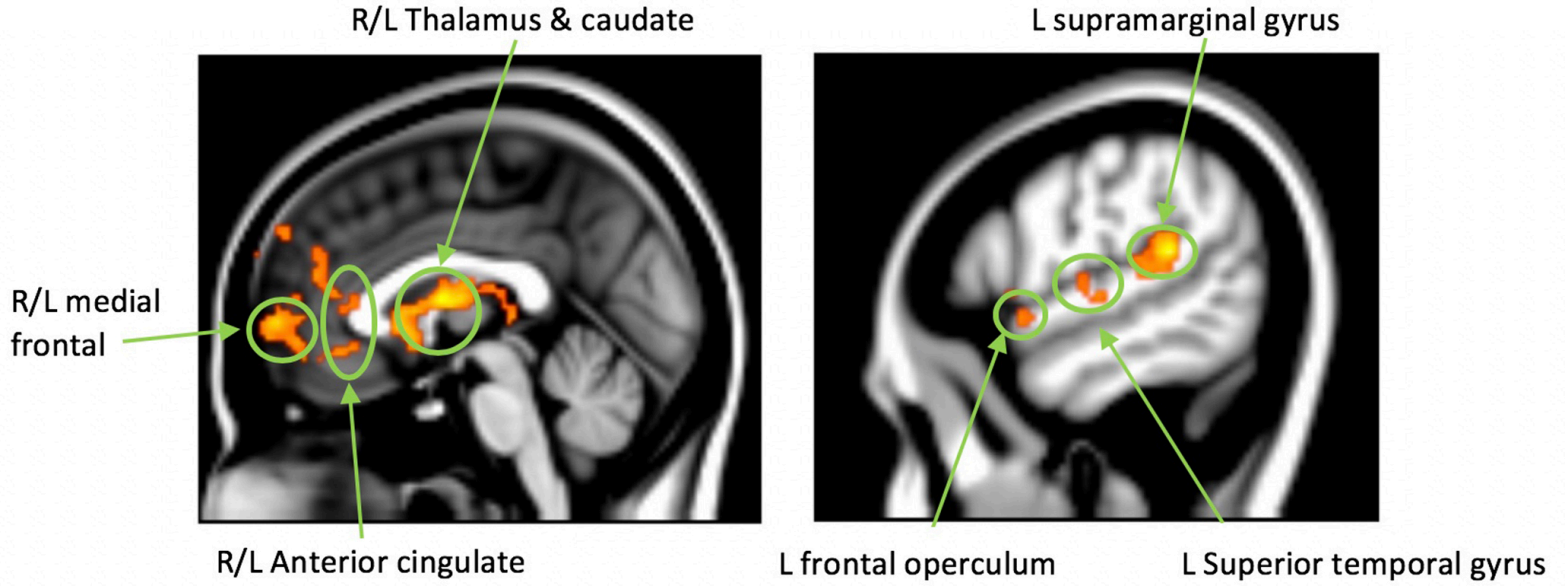
Problem-solving task: increased neural activation post-treatment is associated with improvements in mood lability. **Legend**: While viewing family problems, improvements in mood lability (Children’s Affective Lability Scale) from pre- to post-treatment in participants who received FFT-HR were associated with increased activation in bilateral caudate/thalamus (Max Z = 3.92, *p* <0.001) and bilateral medial prefrontal cortex/anterior cingulate (Max Z = 3.84, *p*<0.001); and in left supramarginal (Max Z = 3.95, *p* <0.002), left frontal pole (Max Z = 3.53, *p* = 0.04), and left frontal opercular (Max Z = 3.76, *p* = 0.04) regions. No changes in activation were found for those in the EC condition or when participants were viewing or attempting to solve non-familial problems.

Analyses of neural activation during the problem-solving task were conducted with 16 subjects in the FFT-HR group and 20 in the EC group with useable data for this task. At the pre-treatment scan, results from a whole brain voxel-wise analysis, with family-wise error correction for multiple comparisons at the cluster level, showed that imagining and solving family problems were associated with enhanced activity in medial frontal, lateral frontal, and cingulate cortices, regions that play a key role in processes of introspection, emotion regulation, and conflict resolution. In a whole brain voxel-wise analysis comparing pre- to post-treatment changes in activation while participants viewed statements of family problems, the FFT-HR group, but not the EC group, showed treatment-related increases in activation in bilateral caudate/thalamus (Max Z = 3.92, *p*<.001), bilateral medial prefrontal/anterior cingulate (Max Z=3.84, *p*<.001), left supramarginal/superior temporal (Max Z = 3.95, *p*<.002), and left frontal operculum (Max Z = 3.76, *p*<.05) cortices, and left frontal pole (Max Z = 3.53, *p*<.05). When comparing pre- to post-treatment changes while participants imagined solving family problems, increases in activation in the right precuneus (Max Z = 3.75, *p*<.001) were observed. Increases in activation in all the areas noted above were associated with pre- to post-treatment improvements in CALS mood instability scores (Fig. **3**).

Notably, these results were associated with viewing and solving family problems but not viewing or solving non-familial problems. Covarying for age or sites balanced with medication exposures did not change these results. Thus, observed neural changes were specific to salient family-related stimuli and corresponded with improvements in mood instability.

## 4 DISCUSSION

In this article, we have presented clinical, psychosocial, and neuroimaging results from one of the first randomized trials of a psychosocial intervention in the early stages of BD. Following phenomenological research on offspring of parents with BD [2-4, 63], we examined a sample of children and teens with a family history of BD I or II, approximately 40% of whom had otherwise specified BD and 60% of whom had a major depressive disorder. Interestingly, about 25-30% of high-risk youth in this study [57] and in the Course and Outcome of Bipolar Youth study [58] showed clinical remission over time. Long-term remission in young adulthood was also observed in an 8-year follow-up of prepubertal and early adolescent children who presented with full manic or mixed episodes [64]. The reasons why some youth with bipolar spectrum disorders fully recover while others have ongoing mood symptoms and psychosocial impairment are unclear.

Our results suggest that in high-risk youth, a brief family psychoeducational therapy is effective in reducing the likelihood of new depressive episodes and suicidal ideation or behaviors over an average of 2 years after a period of mood symptoms. We identified possible psychosocial and neural mediating mechanisms of these treatment effects. First, the youth’s view of family functioning, such as whether the mother/child relationship is characterized by high or low conflict, is an important mediator of whether children show improvements in depressive symptoms or suicidal ideation/behavior in FFT-HR compared to EC. Whether it is critical to work directly with the family to achieve these changes in youth’s perceptions or whether such changes can emerge from individual cognitive restructuring or acceptance-based interventions deserves investigation.

The neuroimaging results have implications for understanding brain mechanisms in high-risk youth at rest while processing emotions and while attempting to regulate emotions during problem-solving, as well as the ways in which these functions change with psychosocial intervention. Resting-state findings indicate that compared to EC, FFT-HR was associated with stronger connectivity between the VLPFC and aDMN, possibly indicating enhanced self-awareness, illness awareness, and emotion regulation. Stronger connectivity was associated with improvements in mood lability on the parent-rated CALS. FFT-HR was also associated with increased anterior DMN connectivity, which was correlated with pre- to post-treatment improvements in Children’s Depression Rating Scale scores. DMN connectivity, in concert with enhanced prefrontal connectivity, may reflect adaptive changes in neural networks indicative of “neural reserve” or the capacity to tolerate brain insults [65, 66].

Changes in neural networks in youth at risk for BD are observable at rest prior to symptom onset, as demonstrated by previous findings that VLPFC-caudate dysconnectivity correlates with family chaos and amygdala-fusiform dysconnectivity correlates with family rigidity in healthy offspring of parents with BD, compared to offspring of healthy parents or offspring of parents with depression [8, 56]. Specifically, healthy offspring of bipolar parents show stronger network connectivity between the VLPFC and executive control regions, as well as increased prefrontal-to-limbic connections at rest compared to other healthy comparison groups. In contrast, symptomatic high-risk youth show stronger VLPFC-aDMN connectivity compared to healthy control offspring. After exposure to FFT, this VLPFC-aDMN connectivity increases further in strength, and may mediate the association between FFT-HR and depression improvement after mood symptoms in high-risk offspring have begun [46].

We observed that during the emotion processing task, DLPFC activation increased from pre- to post-treatment in the FFT-HR group but not in the EC group. These increases were also correlated with improvement in depressive symptoms, while pre/post decreases in amygdala/hippocampal activation were correlated with improvement in hypo/mania symptoms. In the family situations problem-solving task, activation in the medial frontal cortex increased from pre- to post-treatment in the FFT-HR group more than in the EC group, and was correlated with improvements in mood instability. Thus, across tasks, psychosocial treatment was associated with the enhancement of emotion regulation circuitry, although the specific location within the frontal cortex differed based on task design and demands.

Our findings indicate changes during treatment in corticolimbic networks that promote emotion regulation, self-reflection, and awareness. These changes correlate with improvements in symptoms and behavior, and they occur to a greater extent in FFT-HR than in standard psychoeducation. Together, these neuroimaging findings across rest and task-based fMRI suggest that the change mechanisms of FFT-HR and EC may be separable, with FFT-HR having a greater association with executive regulatory control through the prefrontal cortex, commensurate with the teaching of communication and problem-solving skills in FFT-HR that promote these functions.

### 4.1 Study Limitations

First, the sample sizes for the neuroimaging analyses were modest. Nonetheless, we found robust and consistent results across resting and task-based fMRI after correcting for multiple comparisons and adjusting for neuroimaging site, age, and sex. Second, high-risk youth were clinically heterogeneous, and were on a variety of medication regimens, with random assignment stratified on whether the participant was taking a mood stabilizing or antipsychotic agent or neither. HR youth had primary and comorbid diagnoses that are typical of bipolar offspring samples, who are frequently treated with a combination of psychosocial and pharmacological interventions. The treatment groups were not significantly different in distributions of diagnoses or medication exposure at baseline or follow-up, and univariate analyses did not find that primary diagnosis or medication class predicted treatment-related differences in the neuroimaging results.

Third, the EC condition was matched to the FFT-HR condition in duration (4 months) but not in the number of sessions (12 *vs*. 6). Group differences in symptom outcomes or neural activation could have been due to nonspecific differences in the number of clinical contacts. A survival analytic model indicated the effects of the treatment group but not the number of sessions on time to new mood episodes. Further, the identification of neural networks relevant to the skills targeted in FFT-HR and their association with symptom changes make this possibility less likely. We recognize the importance of replication studies that match these intervention groups on session frequency as well as the duration of treatment to determine whether treatment content is more essential than treatment intensity.

Finally, we could not disentangle the specific components of FFT-HR that map onto changes in psychosocial or neural variables. Within-person changes resulting from psychoeducation (*e.g*., greater insight into illness) are likely to have different neurocognitive correlates than changes resulting from communication skills training. Future studies should examine, for example, whether psychoeducation strengthens cognitive control or whether communication skills training improves language network connectivity.

## CONCLUSION

What have we learned about high-risk states in BD? First, working with families of high-risk youth on their understanding of the child’s mood swings and their ability to communicate and solve problems may be associated with a more favorable trajectory of mood symptoms and reductions in suicidal ideation and behavior. The effects of family intervention in the high-risk period appear to be more consistently focused on depression than on hypomania or mania, possibly reflecting the dominant polarity of symptoms early in the course of the disorder [67].

FFT-HR appears to enhance neural circuits that underlie emotion regulation, such as activity in prefrontal and executive control regions critical for adaptive self-regulation, especially in the context of the significant and known family environmental disturbances associated with bipolar illness [10, 13, 68]. It also enhances the child’s perceptions of the support and cohesion available within the family unit, which in turn may motivate the proband to regulate their own expression of negative emotion. In the psychiatric literature, the importance of perceived social support in protecting vulnerable individuals from episodes of depression, together with the individual’s intrinsic capacities to adapt to stress, cannot be overemphasized [69-71].

We were unable to show that FFT-HR is effective in preventing the onset of syndromal BD I or II. Although FFT-HR had preventative effects on depressive episodes, the incidence of conversions to bipolar I or II, and more generally of new onset manic episodes, was relatively low (16%) over the short period of follow-up (average 2 years). Studies in which high-risk youth are followed over longer periods may demonstrate the preventative effects of family or other psychosocial interventions. Other approaches to early intervention that emphasize skill-building, such as dialectical behavior therapy [72], interpersonal and social rhythm therapy [73], or mindfulness-based cognitive therapy [74], may also provide powerful and potentially neuroplastic effects in the early stages of bipolar illness. Finally, the preventative efficacy of family psychoeducational interventions for the onset of disorders other than BD, such as schizophrenia or recurrent major depressive disorder, deserves investigation in clinical trials.

## Figures and Tables

**Table 1:** Youth at high-risk for bipolar disorder: participant characteristics.

**Variable**	**Family-focused Therapy ** **(n = 61)**		**Enhanced Care** **(n = 66)**		**Total (N = 127)**		**Range**
	Mean	SD	Mean	SD	Mean	SD	
Age, years	13.2	2.7	13.3	2.5	13.2	2.6	9.0-17.8
Socioeconomic status, (M, SD) (class 1-5)^a^	3.7	0.8	4.1	0.8	3.9	0.8	1-5
Young Mania Rating Scale, baseline	12.8	6.8	12.5	7.7	12.6	7.3	0-33
Children's Depression Rating Scale-Revised, baseline	46.3	13.5	48.3	15.5	47.3	14.5	20-88
Children’s Global Assessment Scale, last 2 weeks, baseline	52.7	9.8	52.2	22.5	52.5	10.6	28-80
Adolescent Longitudinal Interval Follow-up Evaluation Psychiatric Status Ratings of Depression, baseline^b^	3.7	1.0	3.9	1.0	3.8	1.0	1.2-6.0
Adolescent Longitudinal Interval Follow-up Evaluation Psychiatric Status Ratings of Hypo/ Mania, baseline^b^	1.6	0.7	1.6	0.7	1.6	0.7	1.0-3.7
**-**	**N**	**%**	**N**	**%**	**N**	**%**	-
Female sex	37	60.7	45	68.2	82	64.6	-
Race, non-white	12	19.7	10	15.2	22	17.3	-
Hispanic ethnicity	15	24.6	8	12.1	23	18.1	-
Major depressive disorder	37	60.7	38	57.6	75	59.1	-
Otherwise specified bipolar disorder	24	39.3	28	42.4	52	40.9	-
**Comorbid Disorders^c^**							
None	6	9.8	11	16.7	17	13.4	-
Internalizing disorders only	21	34.4	26	39.4	47	37.0	-
Externalizing disorders	13	21.3	14	21.2	27	21.3	-
Internalizing and externalizing disorders	21	34.4	15	22.7	36	28.4	-
**Baseline Medications**							
None	23	37.7	33	50.0	56	44.1	-
Lithium	1	1.6	0	0.0	1	0.8	-
Antipsychotic	13	21.3	17	25.8	30	23.6	-
Anticonvulsant	10	16.4	8	12.1	18	14.2	-
Antidepressant	27	44.3	20	30.3	47	37.0	-
Anxiolytic	2	3.3	2	3.0	4	3.1	-
Psychostimulant/other ADHD agent	12	19.7	14	21.2	26	20.5	-

**Notes: **^a^Higher class values for socioeconomic status indicate higher educational level and occupation. ^b^Psychiatric Status Ratings for depression are applied weekly and can range from 1 (asymptomatic) to 6 (fully syndromal, severe). Weekly ratings for hypo/mania range from 1 (asymptomatic) to 8 (syndromal mania, extremely severe), with scores of 6 indicating syndromal hypomania. Scores above are calculated as the mean of each of the 18 weekly ratings prior to random assignment to treatments. ^c^Internalizing disorders include anxiety disorders, eating disorders, and elimination disorders. Externalizing disorders include attention deficit hyperactivity disorder, oppositional defiant disorder, conduct disorder, and disruptive mood dysregulation disorder.

**Table 2:** Youth at high-risk for bipolar disorder: characteristics of 34 participants with pre- and post-treatment neuroimaging scans.

**Variable**	**Family-focused Therapy ** **(n = 16)**		**Enhanced Care ** **(n = 18)**		**Total (N = 34)**		**Range**
	**Mean**	**SD**	**Mean**	**SD**	**Mean**	**SD**	
Age, years	14.6	2.4	13.5	2.8	14.0	2.6	9.0-17.8
Socioeconomic status, (M, SD) (class 1-5)^a^	3.8	0.4	4.1	0.7	3.9	0.6	3-5
Young Mania Rating Scale, baseline	10.0	7.7	11.8	6.2	10.9	6.9	0-28
Children's Depression Rating Scale-Revised, baseline	46.9	10.3	50.7	17.7	48.9	14.6	27-88
Children’s Global Assessment Scale, last 2 weeks, baseline	57.3	8.2	54.3	10.2	55.9	9.1	44-71
Adolescent Longitudinal Interval Follow-up Evaluation Psychiatric Status Ratings of Depression, baseline^b^	3.8	0.9	3.8	1.1	3.8	1.0	1.9-5.4
Adolescent Longitudinal Interval Follow-up Evaluation Psychiatric Status Ratings of Hypo/Mania, baseline^b^	1.5	0.7	1.5	0.6	1.5	0.6	1.0-3.1
	**N**	**%**	**N**	**%**	**N**	**%**	**-**
Scanning site Stanford UCLA	7 9	43.8 56.3	10 8	55.6 44.4	17 17	50.0 50.0	**-**
Female sex	8	50.0	11	61.1	19	55.9	-
Race, non-white	9	56.3	5	27.8	14	41.2	-
Hispanic ethnicity	3	18.8	1	5.6	4	11.8	-
Major depressive disorder	11	68.8	8	44.4	19	55.9	-
Otherwise specified bipolar disorder	5	31.3	10	55.6	15	44.1	-
**Baseline Medications**							
None	4	25.0	10	55.6	14	41.2	-
Lithium	0	0	0	0	0	0	-
Antipsychotic	3	18.8	7	38.9	10	29.4	-
Anticonvulsant	4	25.0	2	5.9	6	17.7	-
Antidepressant	8	50.0	2	5.9	10	29.4	-
Anxiolytic	0	0	1	3.0	1	2.9	-
Psychostimulant/other ADHD agent	3	18.8	4	22.2	7	20.6	-

**Note:**^a^Higher class values for socioeconomic status indicate the higher educational level and occupation. ^b^Psychiatric Status Ratings for depression are applied weekly and can range from 1 (asymptomatic) to 6 (fully syndromal, severe). Weekly ratings for hypo/mania range from 1 (asymptomatic) to 8 (syndromal mania, extremely severe), with scores of 6 indicating syndromal hypomania. Scores above are calculated as the mean of each of the 18 weekly ratings prior to random assignment to treatments.

## Data Availability

Not applicable.

## LIST OF ABBREVIATIONS

aDMN: Anterior Default Mode Network
BD: Bipolar Disorder
CALS: Children’s Affective Lability Scale
CBQ: Conflict Behavior Questionnaire
DLPFC: Dorsolateral Prefrontal Cortex
EC: Enhanced Care
FFT-HR: Family-Focused Therapy for High-Risk Youth
OSBD: Other Specified Bipolar Disorder (formerly bipolar disorder, not otherwise specified)
PSR: Psychiatric Status Ratings from Adolescent Longitudinal Interval Follow-up Evaluation
VLPFC: Ventrolateral Prefrontal Cortex
